# Risk factors for actinic keratosis, non‐melanoma skin cancer and cutaneous malignant melanoma in persons with and without Parkinson's disease: A cross‐sectional study

**DOI:** 10.1002/ski2.464

**Published:** 2024-10-04

**Authors:** Esther Azizi, Hana Feuerman, Idit Peleg, Felix Pavlotsky, Zvi Segal, Bernice Oberman, Nirit Lev, Emmilia Hodak, Ruth Djaldetti, Sharon Hassin‐Baer, Rivka Inzelberg

**Affiliations:** ^1^ Department of Dermatology Chaim Sheba Medical Center Tel Hashomer Israel; ^2^ Faculty of Medicine & Health Sciences Tel Aviv University Tel Aviv Israel; ^3^ Department of Dermatology Rabin Medical Center—Beilinson Hospital Petah Tikva Israel; ^4^ Department of Internal Medicine D Rabin Medical Center—Beilinson Hospital Petah Tikva Israel; ^5^ Biostatistics and Biomathematical Unit Gertner Institute for Epidemiology & Health Policy Research Chaim Sheba Medical Center Tel Hashomer Israel; ^6^ Department of Neurology Meir Medical Center Kfar Saba Israel; ^7^ Movement Disorder Clinic Rabin Medical Center—Beilinson Hospital Petah Tikva Israel; ^8^ Movement Disorder Institute Chaim Sheba Medical Center Tel Hashomer Israel; ^9^ Department of Neurology and Neurosurgery Sagol School of Neuroscience The Faculty of Medicine & Health Sciences Tel Aviv University Tel Aviv Israel

## Abstract

**Background:**

An epidemiological link between Parkinson's disease (PD) and cutaneous malignant melanoma (CMM) has been widely reported. The role of CMM risk factors in this association is unclear.

**Objectives:**

To compare rates of risk factors for skin tumours, specifically actinic keratosis (AK), non‐melanoma skin cancer (NMSC) and CMM, between persons with and without PD.

**Methods:**

In this cross‐sectional observational study, patients attending tertiary PD clinics and community controls were interviewed for background/medical data and underwent dermatological examination. Logistic regression models assessed risk factors for skin tumours and their interactions with PD status.

**Results:**

Included were 141 persons with PD and 155 controls; mean age 71.7 and 72.6 years, respectively. In both groups, the majority were males of Ashkenazi origin. Altogether, AK, basal cell carcinoma, squamous cell carcinoma and CMM were recorded in 76 (53.9%) persons with PD and 92 (59.3%) controls (NS). CMM prevalence predominated in PD patients. In the PD group, prolonged sun exposure (*p* = 0.007), freckles (*p* < 0.001) and solar lentigines (*p* = 0.008) were associated with skin tumours. In the control group, dysplastic atypical moles were negatively associated with skin tumours (*p* = 0.017). Logistic regression of the whole cohort showed that older age (*p* < 0.001), fair complexion (*p* = 0.04) and prolonged sun exposure (*p* = 0.01) were associated with skin tumours, but PD status was not independently associated, and no interactions were found between PD status and CMM risk factors.

**Conclusions:**

Periodic dermatological screening of PD populations is mandatory, especially for carriers of major phenotypic risk factors or presenting with AK, NMSC or CMM.

## Graphical abstract

Despite reports of epidemiological links, an independent association of Parkinson's disease (PD) with cutaneous malignant melanoma (CMM) remains unclear. We report that CMM predominates among persons with than without PD. A major strength of this study is its comprehensive scope, which entailed evaluating a wider range of skin tumours, including single and multiple pre‐cancerous AK, NMSC and CMM, co‐occurring in individuals with or without PD. Furthermore, it explores the complex relations of well‐established CMM risk factors with these tumours in either group.

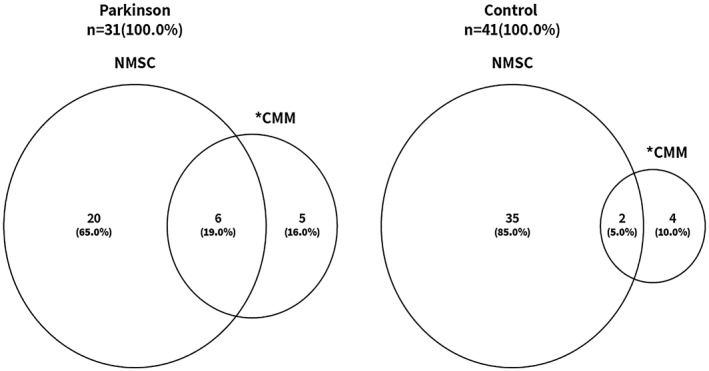

**What is already known about this topic?**

- An epidemiological link has been shown between Parkinson's disease (PD), cutaneous malignant melanoma (CMM) and non‐melanoma skin cancer (NMSC).
- It remains unclear if PD is independently associated with the co‐occurrence of CMM and NMSC or whether it interacts with commonly shared melanoma risk factors.
- Identifying risk factors associated with these skin tumours in the PD population is important for early diagnosis.

**What does this study add?**

- Prolonged sun exposure, freckles and solar lentigines were associated with precancerous actinic keratosis, NMSC and CMM in the PD group, but not in the control group (without PD).
- Total body nevi count was not associated with skin tumours in either group.
- PD status did not associate independently with this outcome or interact with CMM risk factors.

## 1 INTRODUCTION

An epidemiological association between Parkinson’s disease (PD) and cutaneous malignant melanoma (CMM) has been consistently reported.^1^, ^2^, ^3^ Several meta‐analyses have recorded CMM rates as 1.3–3.8 times higher in PD cohorts than in the general population.^4^, ^5^, ^6^ Moreover, PD–CMM co‐occurrence has been observed in first‐ and second‐degree relatives of persons with PD and CMM.^7^, ^8^, ^9^ This relation is bidirectional, as persons with CMM were found to have a 4.2‐fold increased risk of acquiring PD.^10^ Non‐melanoma skin cancer (NMSC, including basal cell carcinoma [BCC] and squamous cell carcinoma [SCC]) also occur significantly more frequently in PD than in non‐PD cohorts, with a relative risk (RR) of 1.2–2.1.^1^, ^11^, ^12^, ^13^, ^14^

The mechanisms underlying the established CMM–PD association are currently unclear. Both diseases target melanocytes, which originate from the neural crest during embryologic development. These cells share common genes and mutations involved in melanin synthesis, cellular differentiation, cell‐cycle regulation, apoptosis, ubiquitination and DNA repair.^15^, ^16^ Genetic predisposition, ageing and external stimuli—particularly exposure to solar ultraviolet radiation (UVR)—may contribute to the degeneration and carcinogenesis of these cells in affected individuals.^17^, ^18^, ^19^

Skin pigmentation significantly affects sun sensitivity and susceptibility to the carcinogenic effect of overexposure to solar UVR. Thus, studying the skin phenotype of PD patients with and without CMM may provide measurable clinical means to identify individuals susceptible to both diseases^19^ and may further clarify the pathogenesis of CMM in PD patients.

In a multi‐centre North American study of 3106 persons with PD, older age (72.6 vs. 68.4 years, *p* < 0.001), fair skin (56.9%), blue eyes (42.0%) and severe or blistering sunburns in childhood (40.9%) were identified as the most common risk factors for CMM among 92 persons with comorbid CMM, compared to those without it.^20^ Similar findings were documented in an Israeli multi‐centre study of 1395 persons with PD: those with CMM (*n* = 20) had higher sun sensitivity (not otherwise specified) (60.0% vs. 30.9%, *p* = 0.0054) and poor tanning ability (45.0% vs. 17.0%, *p* = 0.0036).^21^ Neither study found a significant difference in levodopa exposure between the groups. A moderate or higher degree of sun sensitivity was also reported in a single case‐control study of 150 Portuguese persons with PD, compared to 146 age‐matched controls without PD (32.7% vs. 24.7%; statistical significance was not evaluated).^22^ In this Portuguese cohort, the overall frequency of neoplastic skin tumours, including actinic keratosis (AK) and BCC, was higher in the PD group (23.0% vs. 13.7%; OR 1.92, 95% CI 1.05–3.51).

AK and NMSC, the hallmarks of sun‐induced carcinogenesis,^23^ have been cited as the strongest risk factors for CMM (RR 4.28, 95% CI 2.80–6.55). Additional risk factors are solar lentigines and elastosis, fair versus dark skin colour, blue versus dark eye colour, red versus dark hair colour, freckles and a family history of CMM. The respective RRs cited^24^ were 2.02, 95% CI 1.24–3.29; 2.06, 95% CI 1.68–2.52; 1.47, 95% CI 1.28–1.69; 1.47, 95% CI 1.28–1.69; 2.10, 95% CI: 1.80–2.45 and 1.74, 95% CI 1.41–2.14.

A comparative evaluation of these commonly shared CMM risk factors among PD patients and controls, with and without a wide range of skin tumours, may further clarify whether PD is independently associated with AK, NMSC and CMM, or interacts with any of these risk factors, particularly overexposure to solar UVR.

The aim of this study was to compare the prevalence of major phenotypical and environment‐related risk factors for CMM, and their associations with a wider spectrum of skin tumours, specifically with AK, NMSC and CMM, in persons with and without PD.

## 2 MATERIALS AND METHODS

### 2.1 Design

This cross‐sectional observational study was conducted in the movement disorders and PD outpatient clinics of the Chaim Sheba and Rabin Medical Centres in Israel. The study was approved by the institutional ethics committees of the two medical centres (approval numbers 4046‐17‐SMC and 0792‐15‐RMC, respectively). Participation was voluntary and all participants provided written informed consent.

### 2.2 Participants

Persons with PD who attended the biannual meeting of the Israel Parkinson Association in September 2015 were invited to participate in this study, which constituted part of the MD thesis of one of the authors (I.P.) (Pigmented Skin Lesions and Melanoma Risk Factors in Patients with Parkinson Disease, MD thesis, Faculty of Medicine and Health Sciences, Tel Aviv University, March 2018). In addition, consecutive patients with PD who attended the movement disorders and PD outpatient clinics at the Chaim Sheba and Rabin Medical Centres between November 2016 and January 2021 were offered the opportunity to participate in the study. Only those patients who met the UK PD Society Brain Bank's neurological criteria^25^ for idiopathic PD were included.

For the control group, people without PD who were similar in age, gender and ethnicity were recruited in a number of ways. First, participants with PD were asked to suggest one person from their social circle (e.g., a spouse or a friend). To mitigate potential biases, priority was given to PD spouses, followed by friend/s. Persons attending community day centres for older adults in central Israel were also invited. Finally, persons attending the institute for annual medical screening at the Chaim Sheba Medical Center, which follows healthy persons for systemic disease prevention, were invited to join the study.

Study inclusion criteria were as follows: agreement to respond to the questionnaire addressing his/her medical background and sun exposure habits and to undergo complete skin examination by a senior female dermatologist. Participation was voluntary and all participants provided written informed consent.

### 2.3 Procedure

The background and clinical information for each study participant was obtained through a personal interview using a pre‐coded questionnaire. For persons with PD the data included were age at diagnosis of PD and details of anti‐Parkinsonian medications. For the whole cohort, the collected data included demographics, past medical history, family history of PD and skin cancer including CMM and NMSC, a personal history of documented CMM/NMSC and details regarding sun exposure habits and responses. Sun sensitivity was described in terms of three levels, low sensitivity was defined as no sunburn after the first exposure in the summer, brown tanning (skin phototype ≥ III) and no history of sunburn episodes; intermediate sensitivity was defined as a combination of low and high sensitivity and high sensitivity was defined as always acquiring a sunburn after the first exposure in the summer, no tanning ability (skin phototype I) and a history of previous sunburn episodes. Prolonged sun exposure was categorised by context, as level 0 (none), level 1 (one of the following: recreational, occupational or unspecified) and level 2 (two or more of the following: recreational, occupational or unspecified). Sunscreen use was categorised as never, rarely or sometimes/always.

All participants underwent a systematic whole‐body dermatological examination, including dermoscopy, conducted exclusively by the same two senior dermatologists (E.A. or H.F., both principal investigators in the study). These examinations adhered to a predefined protocol in order to ensure consistency across sites and between dermatologists. Inter‐observer biases were controlled by repeated cross examinations of a sample of participants at each location. Both dermatologists used the Waldmann diagnostic hand light 111 with an 11 W compact fluorescent lamp as a unified light source, to ensure similar lighting conditions at each location. Skin complexion was categorised as ‘dark’ (lightly pigmented skin and brown/dark hair and dark eye colour), ‘combination of fair and dark’ or ‘fair’ (fair skin, blond/red hair and light eye colour). Evaluation of pigmented lesions included the presence of freckles and solar lentigines (yes/no); total body nevi count (TBNC) > 2 mm in diameter (none, 1–20 and ≥21) and total body count of dysplastic/atypical (Clark's) nevi (DN), based on dermoscopic diagnostic criteria.^26^, ^27^ Suspected skin tumours (AK, BCC, SCC and CMM of any subtype) were documented. Each participant was provided with a written medical report on the clinical and pathological findings for further management and follow‐up, as necessary.

### 2.4 Outcomes

The principal outcome measure, for both PD and control groups, as well as for the entire cohort, was the frequency of risk factors associated with skin tumours.

### 2.5 Statistical analyses

Distributions of demographic and phenotypic variables, sun exposure habits and dermatological findings were compared between persons with and without PD and between those with and without skin tumours. To help protect against multicollinearity, phenotypic variables were combined into index variables.

Continuous variables that were normally distributed were compared using *t*‐tests, adjusting if necessary for inequality of variances. Categorical variables were compared using the chi‐squared or Fisher's exact test, according to sample size. Univariate unconditional logistic regressions were performed for persons with and without skin tumours both within the PD and control groups and over the entire cohort. In the latter analysis, PD status (PD/control) was a covariate. Logistic regression was used due to the dichotomous nature of the outcomes. Variables found to be significant in univariate analysis were entered into multiple logistic regression models using a stepwise method. The stepwise method that we employed (backwards elimination) was used for model validation, ensuring that variables of importance, according to the Akaike Information Criterion remained in the model. Also tested were interactions between the PD status and the variables; they were found to be significant in the multiple logistic regression model. All analyses were conducted using R software (version 3.4.1).^28^

## 3 RESULTS

### 3.1 Background and demographic characteristics

The study cohort comprised 141 persons with PD and 155 without PD (control group). Among the control group, 104 (67.1%) had been recommended by participants in the PD group, 20 (12.9%) were attendees of community day‐care centres and 31 (20.0%) were healthy persons undergoing routine medical screening. The mean ages of the PD and control group members were 71.7 ± 7.3 and 72.6 ± 7.3 years, respectively (NS). Males predominated in both groups (61.7% and 51.6%, respectively, NS). Most participants were of Ashkenazi Jewish origin (71.6% and 76.1%, respectively, NS) (Table 1). The mean duration of PD was 10.6 ± 7.2 years, and the mean duration of levodopa use was 7.4 ± 6.9 years. A family history of PD was more common in the PD group (17.0% vs. 7.1%, *p* = 0.014; OR 2.69, 95% CI 1.26–5.71). The proportions of participants with a family history of CMM or NMSC were similar in the two groups (13.5% and 14.2%, respectively, NS).

**TABLE 1:** Distribution of the demographic and background variables.

Variable	Total	Persons with PD	Persons without PD	*p*‐value
	296 (100)	141 (100)	155 (100)	
	*n* (%)	*n* (%)	*n* (%)	
Age—years				0.3
Mean ± SD	72.2 ± 7.3	71.7 ± 7.3	72.6 ± 7.3	
Gender				0.1
Male	167 (56)	87 (62)	80 (52)	
Female	129 (44)	54 (38)	75 (48)	
Origin				0.4
Ashkenazi	219 (74)	101 (72)	118 (76)	
Non‐Ashkenazi	77 (26)	40 (28)	37 (24)	
PD in family				0.01
No	261 (88)	117 (83)	144 (93)	
Yes	35 (12)	24 (17)	11 (7)	
CMM/skin cancer in family				0.9
No	255 (86)	122 (87)	133 (86)	
Yes	41 (14)	19 (13)	22 (14)	

Abbreviations: CMM, cutaneous malignant melanoma; PD, Parkinson's disease.

### 3.2 Frequency of skin tumours

Overall, one or more than one past and current skin tumours (AK, NMSC and CMM) were recorded in 76/141 (53.9%) persons with PD and in 92/155 (59.3%) controls (NS).

### 3.3 Past personal history of skin tumours

**FIGURE 1:**
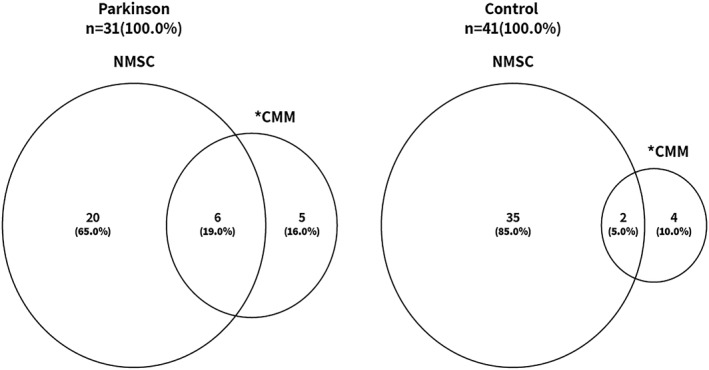
Personal history of ≥1 CMM/NMSC. *CMM rates—35.0% in the Parkinson's disease group versus 15.0% in the control group, *p* = 0.05. CMM, cutaneous malignant melanoma; NMSC, non‐melanoma skin cancer.

A past personal history of CMM or NMSC was documented in 22.0% (31/141) of persons with PD and in 26.5% (41/155) of the control group. CMM was more common in the PD group (11/31 [35.0%]) than in the control group (6/41 [15.0%]), *p* = 0.05. NMSC was less common in the PD group (20/31 [65.0%]) than in the control group (35/41 [85.0%]), NS. Both NMSC and CMM were recorded in 6/31 (19%) persons with PD and in 2/41 (5%) of the controls (Figure 1). Among those with NMSC, 8/20 (40%) of PD patients and 18/35 (51%) of the controls had more than one tumour. Among those with more than one CMM, the corresponding rates were 2/11 (18.8%) and 0 (NS).

### 3.4 Currently diagnosed skin tumours

In the dermatological examination performed for this study, the investigators diagnosed one or more new skin tumours in 45/141 (31.9%) of those with PD and in 51/155 (32.9%) of the control group. The respective proportions of participants with AK were 41.1% and 47.7%; with BCC, 10.6% and 13.5%; with SCC, 5.7% and 10.3% and with CMM, 6.4% and 1.9%, the latter yielding to an OR of 3.45 (95% CI 0.92, 13.0) (NS for all comparisons) (Table 2).

**TABLE 2:** Frequency of skin tumours in 296 persons with and without PD.

Skin tumours	Persons with PD	Persons without PD	Univariate OR (95% CI)^a^
	*N* = 141	*N* = 155	
	*n* (%)	*n* (%)	
Personal history of ≥1 CMM/NMSC	31 (22.0)	41 (26.5)	0.78 (0.46, 1.34)
Currently diagnosed with ≥1 skin tumours			
AK	58 (41.1)	74 (47.7)	0.76 (0.48, 1.21)
BCC	15 (10.6)	21 (13.5)	0.76 (0.38, 1.54)
SCC	8 (5.7)	16 (10.3)	0.52 (0.22, 1.26)
CMM	9 (6.4)	3 (1.9)	3.45 (0.92, 13.03)
Total^b^	76 (53.9)	92 (59.4)	0.8 (0.5, 1.27)

Abbreviations: AK, actinic keratosis; BCC, basal cell carcinoma; CI, confidence interval; CMM, cutaneous malignant melanoma; NMSC, non‐melanoma skin cancer; OR, odds ratio; SCC, squamous cell carcinoma; PD, Parkinson's disease.

^a^ The reference category for the ORs is ‘no skin tumour’.

^b^ Some individuals had more than one tumour of a given type.

Among those with precancerous AK, the rates of currently diagnosed BCC were significantly higher, compared to those without AK, in both groups: 22.4% versus 2.4% among persons with PD (*p* < 0.001) and 20.3% versus 7.4% among controls (*p* = 0.04). Higher rates of SCC, co‐occurring with AK, were noted only among controls—16.2% versus 4.9% in those without AK (*p* = 0.04). No significant changes were noted in the CMM rates between those with or without AK in either group: 6.9% versus 6.0% among PD persons (*p* = 0.4) and 2.7% versus 1.2%, respectively, among controls (*p* = 0.2). Past history of any skin cancer was also significantly associated with the presence of AK in either group: 36.2% versus 12% among PD (*p* < 0.001) and 37.8% versus 16%, respectively, among controls (*p* = 0.004) (data not presented in tables).

### 3.5 The frequency of skin tumours by demographic, phenotypic and sun exposure variables

**FIGURE 2:**
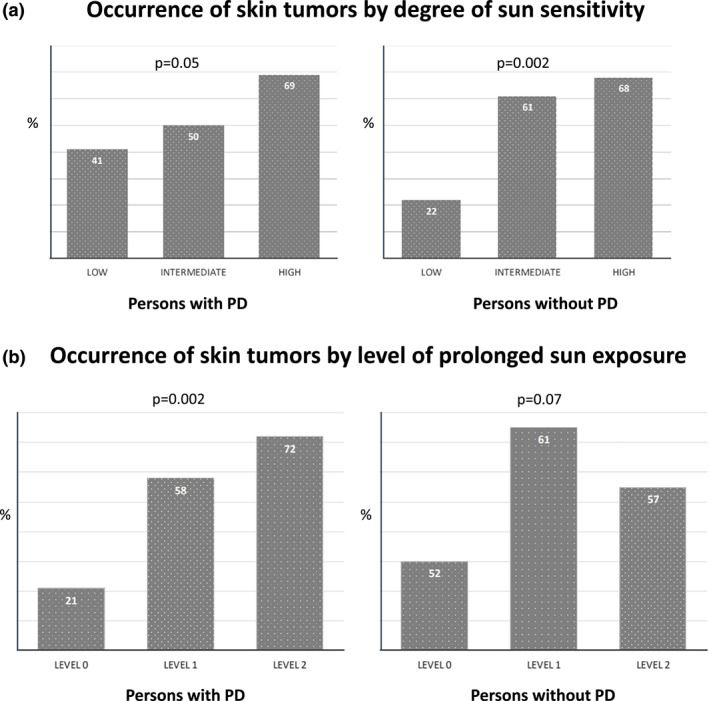
(a) The dose‐response‐like relationship between the degree of sun sensitivity (low to high) and skin tumours in the PD (*p* = 0.05) and control groups (*p* = 0.002). Numbers indicate percentages with skin tumours in each category. (b) Increasing levels of prolonged sun exposure (0–2) was significantly associated with skin tumours in a dose–response‐like pattern only in the PD group *p* = 0.002. Numbers indicate percentages with tumour in each category. PD, Parkinson's disease.

Tables 3 and 4 present the demographic, phenotypic and sun exposure habits and response that characterise participants with and without one or more recorded skin tumours, in the PD and the control groups, as well as for the entire cohort. For both groups, univariate analysis showed associations of the following characteristics with higher frequency of skin tumours: increasing age, Ashkenazi origin, skin complexion (in a dose–response‐like pattern from combined ‘fair‐dark’ to ‘fair’) and sun sensitivity (in a dose–response‐like pattern from low to high) (Figure 2a). In the PD group only, associations with skin tumours were observed for increasing levels of prolonged sun exposure (in a dose–response‐like pattern) (OR 5.1, 95% CI 1.7–19.1 and *p* = 0.007 for level 1) (Figure 2b), freckles (OR 3.9, 95% CI 1.75–8.85 and *p* < 0.001) and solar lentigines (OR 4.3, 95% CI 1.45–12.5 and *p* = 0.008). In a multivariate logistic regression analysis of the PD group, which controlled for potential risk factors, the presence of freckles was found to be significantly associated with skin tumours (OR 3.13, 95% CI 1.11, 9.53) (data not shown). The TBNC rate was not associated with skin tumours in either group (approximately 92% of both PD and controls had a TBNC of <20 to 0). DN count of 1–15 was more common in the PD group than in the control group (39.0% vs. 20.6%, *p* < 0.001). In the control group DN were negatively associated with skin tumours (OR 0.4, 95% CI 0.2–0.8 and *p* = 0.017).

**TABLE 3:** Frequency of skin tumours by the demographic and phenotypic risk factors of persons with and without PD.

Risk factor	Persons with PD 141 (47.6%)			Persons without PD 155 (52.4%)			Total cohort 296 (100.0%)		
	Skin tumour			Skin tumour			Skin tumour		
	Yes	No	OR (95% CI)	Yes	No	OR (95% CI)	Yes	No	OR (95% CI)
	*n* (%)	*n* (%)		*n* (%)	*n* (%)		*n* (%)	*n* (%)	
	76 (53.9%)	65 (46.1%)		92 (59.3%)	63 (40.6%)		168 (57.0%)	128 (43.0%)	
Parkinson's disease									
No							92 (59.4)	63 (40.6)	1
Yes							76 (53.9)	65 (46.1)	0.8 (0.5, 1.3)
Demographic characteristics									
Male	48 (55.2)	39 (44.8)	1	47 (58.7)	33 (41.3)	1	95 (56.9)	72 (43.1)	1
Female	28 (51.9)	26 (48.1)	0.88 (0.4, 1.7)	45 (60.0)	30 (40.1)	1.05 (0.55, 2.0)	73 (56.6)	56 (43.4)	0.99 (0.6, 1.6)
Age (yr) + SD	74.1 ± 6.7	68.9 ± 7.2	1.11^a^(1.05,1.2)	73.9 ± 7.7	70.6 ± 6.2	1.07 (1.0, 1.1)	73.9 ± 7.2	69.8 ± 6.7	1.09 (1.05, 1.1)
Origin									
Non‐Ashkenazi	15 (37.5)	25 (62.5)	1	11 (29.7)	26 (70.3)	1	26 (33.8)	51 (66.2)	1
Ashkenazi	61 (60.4)	40 (39.6)	2.54 (1.2, 5.4)	81 (68.6)	37 (31.4)	5.17 (2.3,11.6)	142 (64.8)	77 (35.2)	3.62 (2.1, 6.3)
Family history of PD									
No	63 (53.8)	54 (46.2)	1	87 (60.4)	57 (39.6)	1	150 (57.5)	111 (42.5)	1
Yes	13 (54.2)	11 (45.8)	1.01 (0.4, 2.45)	5 (45.5)	6 (54.5)	0.55 (0.2, 1.9)	18 (51.4)	17 (48.6)	0.78 (0.4, 1.6)
Family history of CMM/NMSC									
No	66 (54.1)	56 (45.9)	1	75 (56.4)	58 (43.6)	1	141 (55.3)	114 (44.7)	1
Yes	10 (52.6)	9 (47.4)	0.94 (0.4, 2.5)	17 (77.3)	5 (22.7)	2.6 (0.9,2.55)	27 (65.9)	14 (34.1)	1.6 (0.8, 3.2)
Phenotypic characteristics									
Dark	8 (29.6)	19 (70.4)	1	5 (16.7)	25 (83.3)	1	13 (22.8)	44 (77.2)	1
Combined^b^	59 (57.3)	44 (42.7)	3.18 (1.3, 8.3)	63 (68.5)	29 (31.5)	10.9 (4.1,34.8)	122 (62.6)	73 (37.4)	5.7 (2.9, 11.6)
Fair	9 (81.8)	2 (18.2)	10.7 (2.2,81.4)	24 (72.7)	9 (27.3)	13.3 (4.2,50.1)	33 (75.0)	11 (25.0)	10.15 (4.2,26.6)

Abbreviations: CI, confidence interval; CMM, cutaneous malignant melanoma; NMSC, non‐melanoma skin cancer; OR, odds ratio; PD, Parkinson's disease.

^a^ Increase of 1 year.

^b^ Combination of ‘dark’ and ‘fair’.

**TABLE 4:** Frequency of skin tumours by sun exposure variables and pigmented skin lesions in persons with and without PD.

Risk factor	Persons with PD 141 (47.6%)			Persons without PD 155 (52.4%)			Total cohort 296 (100.0%)		
	Skin tumour			Skin tumour			Skin tumour		
	Yes	No	OR (95% CI)	Yes	No	OR (95% CI)	Yes	No	OR (95% CI)
	*n* (%)	*n* (%)		*n* (%)	*n* (%)		*n* (%)	*n* (%)	
	76 (53.9%)	65 (46.1%)		92 (59.3%)	63 (40.6%)		168 (57.0%)	128 (43.0%)	
History of sun exposure and response									
Sun sensitivity^a^									
Low	9 (40.9)	13 (59.1)	1	4 (22.2)	17 (77.8)	1	13 (32.5)	27 (67.5)	1
Intermediate	38 (50.0)	38 (50.0)	1.4 (0.6, 3.9)	40 (60.6)	26 (39.4)	5.4 (1.7, 20.65)	78 (54.9)	64 (45.1)	2.5 (1.2, 5.4)
High	29 (69.0)	13 (31.0)	3.2 (1.1, 9.7)	48 (67.6)	23 (32.4)	7.3 (2.3, 28.1)	77 (68.1)	36 (31.9)	4.4 (2.1, 9.9)
Level of prolonged sun exposure^a^									
0	4 (21.1)	15 (78.9)	1	12 (52.2)	11 (47.8)	1	16 (38.1)	26 (61.9)	1
1	49 (57.6)	36 (42.4)	5.1 (1.7, 19.1)	72 (61.0)	46 (39.0)	1.4 (0.6, 3.5)	121 (59.6)	82 (40.4)	2.4 (1.2, 4.8)
2	18 (72.0)	7 (28.0)	9.6 (2.55, 44.2)	8 (57.1)	6 (42.9)	1.2 (0.3, 4.8)	26 (66.6)	13 (33.3)	3.25 (1.3, 8.3)
Sunscreen use^a^									
Never	57 (59.4)	39 (40.6)	1	40 (61.5)	25 (38.5)	1	97 (60.2)	64 (39.8)	1
Rarely	3 (50.0)	3 (50.0)	0.7 (0.1, 3.9)	10 (58.8)	7 (41.2)	0.9 (0.3, 2.7)	13 (56.5)	10 (43.5)	0.9 (0.4, 2.1)
Always	16 (43.2)	21 (56.8)	0.5 (0.2, 1.2)	42 (57.5)	31 (42.5)	0.85 (0.4, 1.7)	58 (52.7)	52 (47.3)	0.7 (0.45, 1.2)
Pigmented skin lesions									
Freckles									
No	11 (29.7)	26 (70.3)	1	9 (40.9)	13 (59.1)	1	20 (33.9)	39 (66.1)	1
Yes	65 (62.5)	39 (37.5)	3.9 (1.75,8.85)	83 (62.4)	50 (37.6)	2.40 (1.0, 6.1)	148 (62.4)	89 (37.6)	3.2 (1.8, 6.0)
Solar lentigines									
No	5 (25.0)	15 (75.0)	1	4 (33.3)	8 (66.7)	1	9 (28.1)	23 (71.9)	1
Yes	71 (58.7)	50 (41.3)	4.3 (1.45, 12.5)	88 (61.5)	55 (38.5)	3.2 (0.9, 11.1)	159 (60.2)	105 (39.8)	3.9 (1.8, 9.1)
Mole count									
None	27 (58.7)	19 (41.3)	1	33 (67.3)	16 (32.7)	1	60 (63.2)	35 (36.8)	1
≤20	44 (53.0)	39 (47.0)	0.79 (0.4, 1.6)	54 (56.8)	41 (43.2)	0.6 (0.3, 1.3)	98 (55.1)	80 (44.9)	0.7 (0.4, 1.4)
≥21	5 (41.6)	7 (58.3)	0.50 (0.1, 1.8)	5 (45.5)	6 (54.4)	0.40 (0.1, 1.5)	10 (43.5)	13 (56.5)	0.45 (0.2, 1.1)
Any dysplastic nevi									
No	46 (53.5)	40 (46.5)	1	79 (64.2)	49 (35.8)	1	125 (59.8)	84 (40.2)	1
Yes	30 (54.5)	25 (45.5)	1.04 (0.5, 2.1)	13 (40.6)	19 (59.4)	0.4 (0.2, 0.8)	43 (49.4)	44 (50.6)	0.7 (0.4, 1.1)

Abbreviations: CI, confidence interval; OR, odds ratio; PD, Parkinson's disease.

^a^ In the PD group, data were missing on sun sensitivity in 1 person with no skin tumour; on sun exposure level in 12 persons, 5 with skin tumours and 7 without and on sunscreen use in 2 persons with no skin tumour.

Table 5 shows the results of the multivariate regression model in the entire cohort, with PD status included as a cofactor, after adjustment for risk factors was found to be statistically significant in the univariate analysis. The factors found to be associated with skin tumours were older age (OR 1.08, 95% CI 1.04–1.13 and *p* < 0.001), fair versus dark skin complexion (OR 3.48, 95% CI 1.08–11.62 and *p* = 0.04) and more prolonged (level 2) sun exposure (OR 4.07, 95% CI 1.47–11.89 and *p* = 0.01). PD status was not independently associated with skin tumours frequency. In the analysis of the entire cohort, no interactions were observed between PD status and any of the risk factors significantly associated with skin tumours.

**TABLE 5:** OR and 95% CI of risk factors associated with skin tumours in the study cohort.

Risk factors	OR	95% CI	*p*‐value
Age (1 year increase)	1.08	1.04, 1.13	<0.001
Origin Ashkenazi versus other	1.38	0.63, 3.0	0.42
Skin complexion			
Combined^a^ versus dark	2.59	1.02, 6.77	0.05
Fair versus dark	3.48	1.08, 11.62	0.04
Sun sensitivity			
Intermediate versus low	1.73	0.72, 4.25	0.22
High versus low	2.09	0.82, 5.45	0.12
Level of prolonged sun exposure			
1 versus 0	2.8	1.31, 6.13	0.01
2 versus 0	4.07	1.47, 11.89	0.01
Pigmented skin lesions			
Solar lentigines (yes vs. no)	2.21	0.82, 6.17	0.12
Freckles (yes vs. no)	1.22	0.54, 2.73	0.63
PD (yes vs. no)	0.93	0.53, 1.64	0.81

Abbreviations: CI, confidence interval; OR, odds ratio; PD, Parkinson's disease.

^a^ Combination of ‘fair’ and ‘dark’.

## 4 DISCUSSION

The hallmark of the skin tumours recorded in this study cohort is the predominance of CMM in persons with PD, compared to those without it. Previous history of ≥1 CMM was observed in 35% persons with PD versus 15% in controls (*p* = 0.05). Suspected CMM lesions were more commonly detected in the PD than in the control group (6.4% vs. 1.9%, NS).

Yet, the most common tumour in our study, as detected in >40% of the participants in both groups, was AK. Those with precancerous AK had a significantly higher frequency of BCC—in both groups and of SCC—only among the controls.

The co‐occurrence of AK with NMSC or CMM in our study is compatible with a report of 7727 Israelis with PD,^13^ where AK recorded prior to PD diagnosis was strongly associated (*p* < 0.01) with increased risk of BCC (hazard ratio 1.87; 1.70–2.06), as well as of SCC (1.96; 1.90–2.03) and CMM (1.24; 1.08–1.42). The risks of NMSC and CMM were higher when AK appeared at younger ages.

The contrast between the relatively high frequency of AK and relatively low rates of TBNC (in more than 90% of our cohort) is not an unexpected finding. AK and nevi have both been shown to be negatively associated with one another but positively and independently associated with CMM risk.^29^, ^30^, ^31^ Nonetheless, the evidently low TBNC reported here, is compatible with the well‐documented progressive reduction in TBNC with increasing age.^31^, ^32^, ^33^ It may account for the lack of any association of TBNC with skin tumours in general, and CMM in particular, in this study cohort.

The significantly higher frequency of the total DN count (in the range of 1–15) in the PD group than in the control group (39.0% vs. 20.6% and *p* < 0.001) suggests that atypical nevi may be characteristic of PD. This finding, however, may reflect selection bias of the control group. Among patients with PD in a previous study, the rates of more than one large irregular pigmented lesion (which may correspond to DN) was greater in those with CMM (*n* = 20) than in those without it (*n* = 1375): 15.0% versus 2.0%; OR 4.93, 95% CI 0.23–103.63 and *p* = 0.0008.^21^ Similarly, in the Portuguese cohort, higher rates of DN were reported among patients with PD, than in those without it: 1.3% versus 0; OR 4.93, 95% CI 0.23–103.63.^22^ Nevertheless, we found no association of DN, representing a precursor of CMM, with skin tumours among our patients with PD, while in the control group the relative lower rate of DN indeed served as a protective factor.

Upon univariate analysis, increasing levels of prolonged sun exposure, freckles and solar lentigines were found to be associated with skin tumours only among persons with PD. This finding may represent a major pathogenetic role of solar damage in the occurrence of skin tumours among PD patients. Freckles have been found to be associated with a lower minimal solar UVR erythema dose,^34^ suggesting an increased susceptibility to acute sunburn episodes in persons with PD, compared to controls. The dose‐dependent association of increasing levels of prolonged sun exposure and of solar lentigines with skin tumours further suggest a greater susceptibility of persons with PD patients to UVR carcinogenesis following cumulative actinic skin damage.

An unresolved question however relates to the mechanism accounting for the predominance of CMM among patients with PD. Theoretically, future studies focusing on the anatomic distribution of CMM among persons with and without PD, in relation to well‐defined sun‐induced cancerization fields, characterised by multiple evolving pre‐cancerous AK,^35^ may provide some clues to this issue.

In our cohort, the lack of interaction of PD status with any of the examined risk factors excludes a direct link between PD and greater susceptibility to UVR carcinogenesis. Rather, the findings suggest a parallel pathogenesis of two distinct disorders, namely PD and melanocytic or non‐melanocytic skin tumours. Accordingly, melanocytes in the skin and in the substantia nigra may have variable susceptibility to exogenous and endogenous assaults (e.g., accelerated ageing, cumulative solar damage etc.).^2^, ^15^, ^19^, ^36^, ^37^ This hypothesis is supported by the observation that CMM may precede a diagnosis of PD by several decades.^4^, ^20^, ^21^, ^38^, ^39^ Thus, rather than PD itself, the skin of persons with PD—given its germline genetic make‐up and environmental exposure‐related somatic epigenetic changes^2^, ^16^, ^17^, ^18^—may predispose to UVR carcinogenesis.

A major strength of the present study is its comprehensive scope, which entailed evaluation of a wider range of skin tumours, including single and multiple pre‐cancerous AK, NMSC and CMM, co‐occurring in persons with or without PD. Furthermore, it explores the complex relationships between well‐established CMM risk factors and these tumours in both groups. A hypothetical independent association, or interaction of the major risk factors with PD, as a cofactor, was evaluated in the entire cohort. An additional important strength of the study is that unlike previous investigations,^20^, ^21^ it comprised a comprehensive dermatological examination, including dermoscopy, by two senior dermatology experts. Compared to the naked eye,^40^, ^41^, ^42^, ^43^, ^44^, ^45^ this approach provides a more accurate diagnosis of pigmented melanocytic and non‐melanocytic lesions and of CMM in particular,^40^, ^46^, ^47^, ^48^, ^49^ as well as of AK and NMSC. It also spares patients from unwarranted biopsies of benign skin lesions.^49^, ^50^

Limitations of the study include its relatively small number of participants, warranting caution in interpreting the results. Additionally, owing to the cross‐sectional design, pathological confirmation of the diagnosed skin tumours was lacking, as was complementary information on the chronological occurrence of AK, NMSC and CMM, relative to the date of PD diagnosis. The cross‐sectional design may also have affected causal inferences. Another limitation is the fact that some of the control group subjects were recruited through personal referral by a PD patient. The relatively high proportion of Ashkenazi persons in both the PD and the control groups may affect extrapolation of the findings to other ethnic populations.

In conclusion, the absence of unequivocal phenotypic risk markers emphasises the importance of periodic whole‐body dermatological, dermoscopy‐assisted screening of the elderly population for early diagnosis and management of newly evolving skin tumours.^51^ This is of particular relevance to patients with PD and those with AK, NMSC or CMM.

Future studies are needed in order to delineate the subpopulations of PD that may be at risk for CMM/NMSC. Germline genetic testing in PD populations may serve as the basis for deciphering risk alleles, as well as delineations of mutations found in CMM/NMSC, in relation to PD genes. Last but not least is the need to follow longitudinally the evolution and age‐dependent involution of melanocytic nevi and its contribution to CMM risk in persons with PD. The building of a genetic and dermatological database in large populations of PD, with and without CMM/NMSC, may direct us towards common targets for early detection and therapy.

## CONFLICT OF INTEREST STATEMENT

The authors declared no conflicts of interest.

## AUTHOR CONTRIBUTIONS

**Esther Azizi**: Conceptualisation (lead); investigation (lead); methodology (lead); supervision (lead); writing—original draft (lead); writing—review and editing (lead). **Hana Feuerman**: Methodology (equal). **Idit Peleg**: Data curation (equal); methodology (equal). **Felix Pavlotsky**: Data curation (equal); methodology (equal); writing—review and editing (equal). **Zvi Segal**: Project administration (equal); writing—review and editing (equal). **Bernice Oberman**: Methodology (supporting). **Nirit Lev**: Data curation (equal). **Emmilia Hodak**: Methodology (equal); validation (equal); writing—review and editing (equal). **Ruth Djaldetti**: Data curation (equal). **Sharon Hassin‐Baer**: Data curation (equal). **Rivka Inzelberg**: Data curation (equal).

## ETHICS STATEMENT

The study was conducted in adherence to the Helsinki Declaration (2013) and approved by the institutional ethics committees of the Chaim Sheba and Rabin Medical Centres (approval numbers 4046‐17‐SMC and 0792‐15‐RMC, respectively). Participation was voluntary and all the participants provided written informed consent.

## Data Availability

The data supporting this article will be shared upon reasonable request to the corresponding author.
